# Evaluation of Administrative Data for Identifying Maternal Opioid Use at Delivery in Florida

**DOI:** 10.1007/s10995-023-03669-6

**Published:** 2023-05-18

**Authors:** Amanda L. Elmore, Jason L. Salemi, Russell S. Kirby, William M. Sappenfield, Joseph Lowry, Ashley Dixon, Heather Lake-Burger, Jean Paul Tanner

**Affiliations:** 1 Chiles Center, College of Public Health, University of South Florida, 13201 Bruce B. Downs Blvd, MDC56, Tampa, FL 33612-3805 USA; 2 Florida Department of Health, Florida Birth Defects Registry, Tallahassee, USA

**Keywords:** Neonatal abstinence syndrome, Maternal, Opioid, Accuracy, Sensitivity

## Abstract

**Objectives:**

Studies have shown significant increases in the prevalence of maternal opioid use. Most prevalence estimates are based on unverified ICD-10-CM diagnoses. This study determined the accuracy of ICD-10-CM opioid-related diagnosis codes documented during delivery and examined potential associations between maternal/hospital characteristics and diagnosis with an opioid-related code.

**Methods:**

To identify people with prenatal opioid use, we identified a sample of infants born during 2017–2018 in Florida with a NAS related diagnosis code (P96.1) and confirmatory NAS characteristics (N = 460). Delivery records were scanned for opioid-related diagnoses and prenatal opioid use was confirmed through record review. The accuracy of each opioid-related code was measured using positive predictive value (PPV) and sensitivity. Modified Poisson regression was used to calculate adjusted relative risks (aRR) and 95% confidence intervals (CI).

**Results:**

We found the PPV was nearly 100% for all ICD-10-CM opioid-related codes (98.5–100%) and the sensitivity was 65.9%. Non-Hispanic Black mothers were 1.8 times more likely than non-Hispanic white mothers to have a missed opioid-related diagnosis at delivery (aRR:1.80, CI 1.14–2.84). Mothers who delivered at a teaching status hospital were less likely to have a missed opioid-related diagnosis (p < 0.05).

**Conclusions for Practice:**

We observed high accuracy of maternal opioid-related diagnosis codes at delivery. However, our findings suggest that over 30% of mothers with opioid use may not be diagnosed with an opioid-related code at delivery, although their infant had a confirmed NAS diagnosis. This study provides information on the utility and accuracy of ICD-10-CM opioid-related codes at delivery among mothers of infants with NAS.

## Significance

From 2010 to 2017, maternal opioid-related diagnoses at delivery increased by 100% in the US. Most prevalence estimates are based on unverified ICD-10-CM diagnosis codes. Evaluations of maternal opioid-related diagnoses at delivery are extremely limited but essential for utilizing prevalence estimates generated from administrative data.

## Introduction

The opioid crisis has negatively impacted mothers and children, as evidenced by increases in maternal opioid use disorder (OUD) and neonatal abstinence syndrome (NAS) (Haight et al., 2018; Hirai et al., 2021; Ko et al., 2016; Kozhimannil et al., 2020; Leech et al., 2020; Patrick et al., 2015). Opioid use during pregnancy is associated with decreased prenatal care and adverse pregnancy outcomes including postnatal drug withdrawal in the neonate (i.e., NAS), preterm labor, certain birth defects, and stillbirth (Bateman et al., 2021; Maeda et al., 2014; Patrick et al., 2020; The American College of Obstetricians and Gynecologists, 2017; Wen et al., 2021; Whiteman et al., 2014). Prenatal opioid use increases the risk of postpartum opioid overdose (Metz et al., 2016; Whiteman et al., 2014). A recent study of 22 states found that pregnancy-associated mortality involving opioids more than doubled from 2007 to 2016 (Gemmill et al., 2019). The type of maternal opioid use may vary and includes prescribed use for pain management, use during treatment for OUD, misuse of a prescription opioid, and illicit use (Honein et al., 2019; Wexelblatt et al., 2018).

Studies have shown significant increases in the prevalence of maternal opioid use and NAS in the United States (Hirai et al., 2021; Ko et al., 2016; Kozhimannil et al., 2020; Leech et al., 2020; Patrick et al., 2015). From 2010 to 2017, maternal opioid-related diagnoses at delivery and NAS diagnoses at birth increased by 100% or more in 24 states (Hirai et al., 2021). However, most prevalence estimates are based on the presence of unverified *International Classification of Diseases, 10th Revision, Clinical Modification* (*ICD-10-CM*) diagnosis codes (Haight et al., 2018; Hirai et al., 2021; Ko et al., 2016). Whereas recent evaluation studies have found high accuracy of specific ICD-10-CM diagnosis codes for NAS (Elmore et al., 2020; Goyal et al., 2020; Maalouf et al., 2019), complementary analyses of maternal opioid-related codes at delivery are limited. Understanding the sensitivity and accuracy of opioid-related codes at delivery is essential for interpreting and utilizing prevalence estimates generated from administrative data. Previous evaluations among the general population suggest opioid use is underdiagnosed in hospital settings due to a lack of addiction medicine training and perceptions of stigma (Carrell et al., 2015; Howell et al., 2020; Palumbo et al., 2020). However, we lack quantitative data assessing whether the accuracy of assigned opioid-related diagnosis codes differ by maternal and hospital characteristics, particularly among people who use opioids during pregnancy.

To fill this gap in knowledge, the primary aim of this study was to determine the accuracy of ICD-10-CM opioid-related diagnosis codes documented during delivery hospitalization. Secondarily, we aimed to examine the association between maternal and/or hospital level characteristics and likelihood of diagnosis with an opioid-related code at delivery.

## Methods

The Florida Birth Defects Registry conducted a cross-sectional study using a linked data set comprised of Florida vital statistics, hospital discharge data, and abstracted medical record data from infant birth and mother delivery records. To identify people with opioid use during pregnancy, we identified a sample of singleton infants (N = 514) born between 2017 and 2018 in Florida with a diagnosis code indicative of NAS (P96.1) that was assigned during a hospitalization within the first 28 days of life. For the final sample (N = 460), we included only mothers of infants with confirmed NAS based upon a Florida case definition of NAS which has been previously described (Elmore et al., 2020). Mothers who delivered a child with confirmed NAS had their delivery discharge records scanned for diagnosis codes (F11.2X, F11.1X, F11.9X, Z279.891) that could be categorized into “opioid use disorder” (OUD), “opioid use”, or “no opioid-related diagnosis code” in alignment with previous studies (Haight et al., 2018; Hirai et al., 2021). Mothers with ICD-10-CM codes for opioid dependence (F11.20–F11.29) or opioid abuse (F11.10–11.19) were categorized as “OUD”; other mothers diagnosed with ICD-10-CM codes for opioid use (F11.90–F11.99) or long-term use of opiates (Z79.891) were categorized as “opioid use”.

Medical records for the maternal delivery and infant birth hospitalizations were reviewed and abstracted for all mothers of infants with NAS to verify prenatal opioid use through toxicology results, prescription drug data, and reported opioid use. The relationship between maternal characteristics and diagnosis with an opioid-related code was assessed and included maternal age, race/ethnicity, education level, insurance type, and trimester started prenatal care from vital statistics. The delivery hospitals (N = 26) were categorized by number of beds, teaching status and location, and neonatal intensive care unit (NICU) level. Delivery hospital location was determined using the National Center for Health Statistics urban-rural classification scheme (Centers for Disease Control and Prevention, 2017).

Descriptive statistics were calculated to describe the sample by prevalence of any maternal opioid-related codes and by opioid use category. Next, we determined the accuracy of each opioid-related code, as determined by evidence of maternal opioid use in the infant or maternal record, using both the positive predictive value (PPV) and sensitivity metrics. Then, we restricted our sample to mothers with confirmed opioid use (N = 454) to compare the likelihood of receiving an opioid-related diagnosis at delivery across maternal and hospital characteristics. Modified Poisson regression with robust error variance was used to calculate crude and adjusted relative risks (aRR) and 95% confidence intervals (CI) (Zou, 2004). We compared model fit statistics for models with all possible variable combinations and chose the model with the lowest quasi-likelihood under the independence criterion. A sensitivity analysis was conducted to detect within-hospital variation with a generalized linear mixed model. We excluded observations with missing maternal education (N = 8), missing maternal race/ethnicity (N = 1) and “Other” race/ethnicity (N = 5) from our crude and adjusted models due to sample size constraints. Lastly, we assessed other ICD-10-CM drug related codes among the subgroup of mothers without an opioid-related code at delivery. Statistical tests were two-sided and considered significant at p < 0.05. All analyses were conducted in SAS 9.4. This study is exempt from institutional review board approval because the study was deemed an evaluation of public health surveillance methods.

## Results

The sample of mothers of infants with NAS were most commonly ages 25–29 (37.2%), non-Hispanic white (81.3%), with Medicaid insurance (80.9%) (Table 1). The majority of deliveries occurred at a metro/teaching status hospital (59.8%) with at least 500 beds (82.2%). Among the total sample of mothers, 98.7% (N = 454) had confirmed opioid use per medical record review; however, only 65.2% (n = 300) were diagnosed with an opioid-related code at delivery (Table 1). The proportion of mothers diagnosed with an opioid related code that had confirmed opioid use at delivery in the maternal or infant record was nearly 100% for all codes, indicating a PPV of 98.5–100% (Table 1). The sensitivity of the ICD-10-CM opioid-related codes was 65.9%. Among the sample of mothers with confirmed opioid use, 33.7% (N = 155) were not diagnosed with an opioid-related code at delivery. Nearly 50% of mothers in this sub-group were diagnosed with a non-opioid drug related ICD-10-CM code at delivery (Table 2). Of which, 34% were diagnosed with a nicotine related code and 15% were diagnosed with a psychoactive drug related code.

**Table 1:** Sample characteristics and accuracy of ICD-10-CM codes indicative of opioid use at delivery among mothers of infants with confirmed neonatal abstinence syndrome, Florida 2017–2018 (N = 460)

Characteristics	Total sample (N = 460)	Any opioid code^a^ (N = 300)	Opioid use disorder code^b^ (N= 232)	Opioid use code^c^ (N = 68)
Delivery year				
2017	254 (55.2)	153 (51.0)	120 (51.7)	33 (48.5)
2018	206 (44.8)	147 (49.0)	112 (48.3)	35 (51.5)
Age, years				
18–24	61 (13.3)	45 (15.0)	36 (15.5)	9 (13.2)
25–29	171 (37.2)	113 (37.7)	88 (37.9)	25 (36.8)
30–34	143 (31.1)	92 (30.7)	70 (30.2)	22 (32.4)
35+	85 (18.5)	50 (16.7)	38 (16.4)	12 (17.7)
Race/Ethnicity				
Non-Hispanic white	374 (81.3)	243 (81.0)	193 (83.2)	50 (73.5)
Non-Hispanic Black	25 (5.4)	13 (4.3)	8 (3.5)	5 (7.4)
Hispanic^d^	55 (12.0)	41 (13.7)	29 (12.5)	12 (17.7)
Other/unknown/missing^e^	6 (1.3)	3 (1.0)	2 (0.9)	1 (1.5)
Education level				
Some high school	86 (18.7)	57 (19.0)	47 (20.3)	10 (14.7)
High school diploma	193 (42.0)	132 (44.0)	106 (45.7)	26 (38.2)
More than high school	173 (37.6)	105 (35.0)	75 (32.3)	30 (44.1)
Missing	8 (1.7)	6 (2.0)	4 (1.7)	2 (2.9)
Primary payer				
Medicaid	372 (80.9)	250 (83.3)	190 (81.9)	60 (88.2)
Private	67 (14.6)	40 (13.3)	33 (14.2)	7 (10.3)
Self-pay/other/missing	21 (4.6)	10 (3.3)	9 (3.9)	1 (1.5)
Trimester started prenatal care				
First	194 (42.2)	119 (39.7)	89 (38.4)	30 (44.1)
Second, third, or no prenatal care	179 (38.9)	126 (42.0)	97 (41.8)	29 (42.7)
Missing	87 (18.9)	55 (18.3)	46 (19.8)	9 (13.2)
Hospital type^f^				
Metro or nonmetro, non-teaching	185 (40.2)	100 (33.3)	74 (31.9)	26 (38.2)
Metro, teaching	275 (59.8)	200 (66.7)	158 (68.1)	42 (61.8)
Delivery hospital size^g^				
Small or medium	82 (17.8)	34 (11.3)	22 (9.5)	12 (17.7)
Large	378 (82.2)	266 (88.7)	210 (90.5)	56 (82.4)
NICU level				
No NICU, level 1, or level 2	75 (16.3)	32 (10.7)	20 (8.6)	12 (17.7)
Level 3	385 (83.7)	268 (89.3)	212 (91.4)	56 (82.4)
Confirmed drug use	454 (98.7)	299 (99.7)	232 (100)	67 (98.5)
Sources of drug confirmation^h^				
Infant lab	249 (54.1)	173 (57.7)	129 (55.6)	44 (64.7)
Maternal lab	280 (60.9)	191 (63.7)	146 (62.9)	45 (66.2)
Reported history	400 (87.0)	274 (91.3)	211 (91.0)	63 (92.7)
Maternal prescription	301 (65.4)	213 (71.0)	171 (73.7)	42 (61.8)

^a^Any opioid code includes ICD-10-CM codes for opioid dependence (F11.20–F11.29), opioid abuse (F11.10–F11.19), opioid use (F11.90–F11.99) and/or long-term opioid use (Z79.891)

^b^Opioid use disorder code includes ICD-10-CM diagnosis codes for opioid dependence (F11.20–F11.29) or opioid abuse (F11.10–F11.19)

^c^Opioid use code includes ICD-10-CM diagnosis codes for opioid use (F11.9–F11.99) and/or long-term opioid use (Z79.891)

^d^Florida vital statistics categorizes mothers with origins from Spain, Mexico, or Spanish-speaking countries of Central and South America as Hispanic

^e^Other race/ethnicity includes non-Hispanic mothers with a reported race of Asian/Pacific Islander, Native American, or “Other”

^f^Metro or non-metro non-teaching category includes only 2 non-metro hospitals

^g^Delivery hospital size determined by number of beds: Small hospital (≤ 100 beds), medium hospital (> 100 beds), large hospital (≥ 500 beds)

^h^Sources of confirmation are not mutually exclusive. Diagnosis code may be confirmed by multiple sources

Among mothers with confirmed opioid use at delivery (N = 441), 34.2% (N = 151) did not receive an opioid related diagnosis. Multivariable Poisson regression showed significant differences in likelihood of an opioid-related diagnosis by year, maternal race/ethnicity, trimester started prenatal care, and delivery hospital type. Mothers who delivered during 2018 were 28% less likely to have a missed opioid-related diagnosis compared to those who delivered in 2017 (aRR: 0.72, 95% CI 0.55–0.93) (Table 3). Non-Hispanic Black mothers were 1.8 times more likely than non-Hispanic white mothers to have a missed opioid-related diagnosis at delivery (aRR: 1.80, 95% CI 1.14–2.84). Compared to mothers with first trimester prenatal care initiation, mothers who began prenatal care later or had no prenatal care were 28% less likely to have a missed opioid-related diagnosis (aRR: 0.72, 95% CI 0.54–0.96). Lastly, mothers who delivered at a metro, teaching status hospital were 30% less likely (aRR: 0.70, 95% CI 1.01–1.38) to have a missed opioid-related diagnosis than those who delivered at a non-teaching status hospital. Sensitivity analysis to detect within hospital level variation of maternal opioid-related diagnosis was insignificant.

**Table 2:** Documented ICD-10-CM drug related codes among mothers of infants with NAS who did not receive an opioid-related diagnosis at delivery, Florida 2017–2018 (N = 155)

All other drug related codes^a,b^	75 (48.4)
Nicotine	52 (33.5)
Psychoactive	23 (14.8)
Cocaine	9 (5.8)
Cannabis	7 (4.5)
Benzodiazepine or barbiturates	2 (1.3)
Alcohol	1 (0.7)
Stimulants	2 (1.3)
Hallucinogens	1 (0.7)

ICD-10-CM code at delivery indicative of opioid (See Tables 1 and 2)

^a^Drug categories are not mutually exclusive as multiple drug codes could be diagnosed together

^b^Benzodiazepine or barbiturate use (F13.1–F13.99), Alcohol use (F10.1–F10.99), cannabis use (F12.1–F12.99), cocaine use (F14.1–F14.99), stimulant (F15.1–15.99), hallucinogens (F16.1–F16.99), nicotine (F17.1–17.99), psychoactive (F19.1–19.99)

**Table 3:** Comparison of opioid related diagnoses at delivery hospitalization by maternal and hospital characteristics among mothers with confirmed opioid use at delivery, Florida 2017–2018 (N = 441)

Characteristics	No opioid diagnosis code (N = 151)	% No opioid diagnosis	Unadjusted model cRR (95% CI)	Adjusted model aRR (95% CI)^a,b^
Delivery year				
2017	94 (62.3)	39.2	Ref	Ref
2018	57 (37.8)	28.4	0.72 (0.55–0.95)*	0.72 (0.55–0.93)*
Age, years				
18–24	15 (9.9)	25.9	Ref	
25–29	57 (37.8)	33.5	1.30 (0.80–2.10)	1.30 (0.82–2.07)
30–34	47 (31.1)	35.9	1.39 (0.85–2.27)	1.45 (0.90–2.34)
35+	32 (21.2)	39.0	1.51 (0.90–2.52)	1.54 (0.94–2.53)
Race/Ethnicity				
Non-Hispanic white	126 (83.4)	34.8	Ref	Ref
Non-Hispanic Black	12 (8.0)	48.0	1.38 (0.90–2.12)	1.80 (1.14–2.84)*
Hispanic^c^	13 (8.6)	24.1	0.69 (0.42–1.13)	0.83 (0.50–1.37)
Education level				
Some high school	29 (19.2)	34.5	Ref	Ref
High school diploma	60 (39.7)	31.6	0.91 (0.64–1.31)	0.81 (0.58–1.14)
More than high school	62 (41.1)	37.1	1.08 (0.75–1.53)	0.85 (0.60–1.21)
Primary payer				
Medicaid	115 (76.2)	32.2	Ref	Ref
Private	25 (16.6)	39.1	1.21 (0.86–1.71)	1.14 (0.82–1.59)
Self-pay/other/missing	11 (7.3)	55.0	1.71 (1.12–2.61)*	1.60 (1.00-2.56)
Trimester started prenatal care				
First	72 (47.7)	38.5	Ref	Ref
Second, third, or no prenatal care	49 (32.5)	28.7	0.74 (0.55-1.00)	0.72 (0.54–0.96)*
Missing	30 (19.9)	36.1	0.94 (0.67–1.32)	1.01 (0.72–1.41)
Hospital type^d^				
Metro or nonmetro, non-teaching	82 (54.3)	45.3	Ref	Ref
Metro, teaching	69 (45.7)	26.5	0.59 (0.45–0.76)**	0.70 (0.51–0.96)*
Delivery hospital size^e^				
Small or medium	45 (29.8)	57.0	Ref	Ref
Large	106 (70.2)	70.7	0.51 (0.40–0.66)**	0.67 (0.35–1.28)
NICU level				
No NICU, level 1, or level 2	41 (27.2)	56.2	Ref	Ref
Level 3	110 (72.9)	29.9	0.53 (0.41–0.69)**	0.87 (0.46–1.63)

Data are presented as n(%) unless otherwise specified

N = 13 observations were deleted if missing maternal race/ethnicity or education level

*cRR* crude risk ratio; *aRR* adjusted risk ratio; *CI* confidence intervals

^a^Modified Poisson regression model predicting no opioid diagnosis adjusted for delivery year, maternal race/ethnicity, hospital type, hospital bed size, and hospital NICU level based on best model fit criteria

^b^Adjusted model chosen based on lowest QIC

^c^Florida vital statistics categorizes mothers with origins from Spain, Mexico, or Spanish-speaking countries of Central and South America as Hispanic

^d^Metro or non-metro non-teaching category includes only 2 non-metro hospitals

^e^Delivery hospital size determined by number of beds: Small hospital (≤ 100 beds), medium hospital (> 100 beds), large hospital (≥ 500 beds)

*P-value < .05

** P-value < .01

## Discussion

Among mothers who delivered an infant with NAS, we observed high accuracy of maternal opioid-related diagnosis codes at delivery with 99.7% of mothers with an opioid-related code having confirmed opioid use following medical record review. However, our findings suggest a high proportion of mothers with prenatal opioid use may not be diagnosed with opioid related codes at delivery. These findings indicate that reliance on unverified opioid-related codes at delivery may substantially underestimate the prevalence of maternal opioid use and prenatal opioid exposure. We also found significant associations between an opioid-related diagnoses at delivery and birth year, maternal race/ethnicity, prenatal care, and hospital type. This study provides important insight on the utility and accuracy of ICD-10-CM opioid-related codes at delivery hospitalization among mothers of infants with NAS.

The results from our study suggest that maternal opioid use may be severely underreported during delivery hospitalization among mothers of infants with NAS, which is consistent with recent studies. Using administrative data from 4 states including Florida, a study compared the frequencies of mothers with OUD and newborns with NAS and found that in two-thirds of hospitals, the frequency of infants with a diagnostic code indicative of NAS exceeded maternal OUD diagnoses by > 120% (Clark et al., 2021). However, this study included a less sensitive diagnostic code for infant opioid exposure (P04.4) in addition to the diagnostic code indicative of NAS (P96.1). Additionally, a recent population-based study in Ontario, Canada that found reliance on maternal hospital records highly underestimated prenatal opioid use and identified a higher-risk population (Camden et al., 2021). Therefore, the actual prevalence of maternal opioid use may be much higher than previous population-based estimates, and reliance on NAS rates do not fully describe the burden of opioids on pregnant people and children. These findings suggest that surveillance programs relying exclusively on administrative data for identifying maternal opioid use may not be able to adequately plan for targeted prevention efforts and a lack of evidentiary support to request additional resources.

Our study also identified significant maternal and hospital level predictors for receiving an opioid-related diagnosis at delivery. Previous studies have concluded maternal opioid use is most common among non-Hispanic white mothers (Hirai et al., 2021), but our study suggests non-Hispanic Black mothers with confirmed opioid use may be more likely to not receive an opioid-related diagnosis at delivery. This is a novel finding that warrants further investigation, especially given our small sample size of non-Hispanic Black mothers but suggests maternal opioid use may be further underestimated for non-Hispanic Black mothers. A previous study found that Black infants with prenatal opioid exposure were less likely to be diagnosed with NAS than white infants, suggesting there’s racial bias in diagnostic practices for opioid exposed infants (Clark et al., 2021). Additionally, we found that mothers who delivered at non-teaching status hospitals were more likely to have a missed opioid-related diagnosis, suggesting hospital level variation in diagnostic code sensitivity. We also found that mothers with later entry or no prenatal care were more likely to receive an opioid-related diagnosis, which is a noteworthy finding. Potential interpretations may be that pregnant people with later entry or no prenatal care have more severe OUD, a more recent diagnosis of OUD, and/or receive additional screening for drug use during delivery hospitalization. Providers may also be less inclined to document maternal opioid use for pregnant people they’ve provided continuous care to throughout pregnancy due to stigma. Improved care coordination between prenatal providers and delivery clinicians has been noted to improve maternal and infant health outcomes (Patrick et al., 2020). However, given our finding that earlier entry to prenatal care was associated with a lower likelihood of a receiving a diagnosis for opioid use at delivery, it is unclear how strengthened care coordination may influence the sensitivity of maternal opioid-related diagnoses at delivery, which warrants further investigation.

Strengths of this study includes abstraction of both the infant birth and maternal delivery records for confirmation of the NAS diagnosis and maternal opioid use. We also used a statewide Florida sample with 26 hospitals of varying locations and levels of care. We determined two measures of validity for multiple ICD-10-CM opioid-related codes and included hospital level characteristics for regression analyses. Our study is subject to several limitations. First, our sample included mothers of infants with NAS rather than a random sample of all deliveries which limits generalizability of our findings to the general population. The PPV and sensitivity of such codes may be considerably lower if assessed for pregnant people with a wide range of pregnancy outcomes. This limitation is of special concern for those mothers who used opioids near delivery, but their infants did not develop NAS. Also, our sample was selected from births in Florida and therefore our findings may not be generalizable to other states or nationally. We were only able to include opioid-related diagnosis codes from delivery hospitalizations, and a previous analysis reported an 8% under ascertainment of drug abuse codes by relying on the delivery hospitalization relative to all inpatient encounters during pregnancy (Salemi et al., 2020). Additionally, we did not have access to prenatal care records and thus, were only able to confirm maternal opioid use and prenatal exposure by delivery and birth hospitalization records. This limitation may have implications for our association between prenatal care level and likelihood of receiving an opioid-related diagnosis.

## Conclusion

Overall, we found high accuracy of maternal opioid-related diagnoses at delivery. However, our findings suggest that over 30% of mothers with prenatal opioid use may not be diagnosed with an opioid related code at delivery. Additionally, our study found differences in the likelihood of the receipt of an opioid-related code at delivery by maternal and hospital characteristics. Though, it is unclear whether the underdiagnosis of prenatal opioid use is related to quality of care, lack of drug use screening, or a need for hospital coding standardization. Future research is needed to further understand this association overall and by maternal characteristics. Understanding the accuracy of ICD-10-CM codes indicative of maternal opioid use is paramount for public health surveillance. Pregnancy and the postpartum period are critical time frames to identify potentially harmful maternal opioid use to facilitate effective clinical and social interventions.

## Data Availability

Not applicable.

## Code Availability

Upon request from corresponding author.

## References

[CR1] Bateman BT, Hernandez-Diaz S, Straub L, Zhu Y, Gray KJ, Desai RJ, Mogun H, Gawtam N, Huybrechts KF (2021). Association of first trimester prescription opioid use with congenital malformations in the offspring: Population based cohort study. Bmj.

[CR2] Camden A, Ray JG, To T, Gomes T, Bai L, Guttmann A (2021). Identification of prenatal opioid exposure within health administrative databases. Pediatrics.

[CR3] Carrell DS, Cronkite D, Palmer RE, Saunders K, Gross DE, Masters ET, Hyan TR, Von Korff M (2015). Using natural language processing to identify problem usage of prescription opioids. International Journal of Medical Informatics.

[CR4] Centers for Disease Control and Prevention, N. C. (2017). f. H. S. NCHS Urban-rural classification scheme for counties.

[CR5] Clark RRS, French R, Lorch S, O’Rourke K, Rosenbaum KEF, Lake ET (2021). Within-hospital concordance of opioid exposure diagnosis coding in mothers and newborns. Hospital Pediatrics.

[CR6] Elmore AL, Tanner JP, Lowry J, Lake-Burger H, Kirby RS, Hudak ML, Sappenfield WM, Salemi JL (2020). Diagnosis codes and case definitions for neonatal abstinence syndrome. Pediatrics.

[CR7] Gemmill A, Kiang MV, Alexander MJ (2019). Trends in pregnancy-associated mortality involving opioids in the United States, 2007–2016. American Journal of Obstetrics and Gynecology.

[CR8] Goyal S, Saunders KC, Moore CS, Fillo KT, Ko JY, Manning SE, Diop H (2020). Identification of substance-exposed newborns and neonatal abstinence syndrome using ICD-10-CM – 15 hospitals, Massachusetts, 2017. Mmwr. Morbidity and Mortality Weekly Report.

[CR9] Haight SC, Ko JY, Tong VT, Bohm MK, Callaghan WM (2018). Opioid Use Disorder documented at delivery hospitalization - United States, 1999–2014. Mmwr. Morbidity and Mortality Weekly Report.

[CR10] Hirai AH, Ko JY, Owens PL, Stocks C, Patrick SW (2021). Neonatal abstinence syndrome and maternal opioid-related Diagnoses in the US, 2010–2017. Journal of the American Medical Association.

[CR11] Honein MA, Boyle C, Redfield RR (2019). Public health surveillance of prenatal opioid exposure in mothers and infants. Pediatrics.

[CR12] Howell BA, Abel EA, Park D, Edmond SN, Leisch LJ, Becker WC (2020). Validity of incident opioid use disorder (OUD) diagnoses in administrative data: A chart verification study. Journal of General Internal Medicine.

[CR13] Ko JY, Patrick SW, Tong VT, Patel R, Lind JN, Barfield WD (2016). Incidence of neonatal abstinence syndrome – 28 States, 1999–2013. Mmwr. Morbidity and Mortality Weekly Report.

[CR14] Kozhimannil KB, Chantarat T, Ecklund AM, Henning-Smith C, Jones C (2020). Maternal opioid use disorder and neonatal abstinence syndrome among rural US residents, 2007–2014. Journal of Rural Health.

[CR15] Leech AA, Cooper WO, McNeer E, Scott TA, Patrick SW (2020). Neonatal abstinence syndrome in the United States, 2004-16. Health Affairs (Millwood).

[CR16] Maalouf FI, Cooper WO, Stratton SM, Dudley JA, Ko J, Banerji A, Patrick SW (2019). Positive predictive value of administrative data for neonatal abstinence syndrome. Pediatrics.

[CR17] Maeda A, Bateman BT, Clancy CR, Creanga AA, Leffert LR (2014). Opioid abuse and dependence during pregnancy: Temporal trends and obstetrical outcomes. Anesthesiology.

[CR18] Metz TD, Rovner P, Hoffman MC, Allshouse AA, Beckwith KM, Binswanger IA (2016). Maternal deaths from suicide and overdose in Colorado, 2004–2012. Obstetrics and Gynecology.

[CR19] Palumbo SA, Adamson KM, Krishnamurthy S, Manoharan S, Beiler D, Seiwell A, Troiani V (2020). Assessment of probable opioid use disorder using electronic health record documentation. JAMA Network Open.

[CR20] Patrick SW, Davis MM, Lehmann CU, Cooper WO (2015). Increasing incidence and geographic distribution of neonatal abstinence syndrome: United States 2009 to 2012. Journal of Perinatology.

[CR21] Patrick SW, Barfield WD, Poindexter BB (2020). Neonatal opioid withdrawal syndrome. Pediatrics.

[CR22] Salemi JL, Hansen MA, Modak S, Matas JL, Germanos GJ, Raza SA, Agana DFG, Louis JM (2020). Estimating the obstetric co-morbidity burden using administrative data: The impact of the pregnancy-related assessment window. Paediatric and Perinatal Epidemiology.

[CR23] The American College of Obstetricians and Gynecologists (2017). *Opioid Use and Opioid Use Disorder in Pregnancy*. Retrieved from Obstet Gynecol

[CR24] Wen X, Belviso N, Murray E, Lewkowitz AK, Ward KE, Meador KJ (2021). Association of gestational opioid exposure and risk of major and minor congenital malformations. JAMA Network Open.

[CR25] Wexelblatt SL, McAllister JM, Nathan AT, Hall ES (2018). Opioid neonatal abstinence syndrome: An overview. Clinical Pharmacology And Therapeutics.

[CR26] Whiteman VE, Salemi JL, Mogos MF, Cain MA, Aliyu MH, Salihu HM (2014). Maternal opioid drug use during pregnancy and its impact on perinatal morbidity, mortality, and the costs of medical care in the United States. Journal of  Pregnancy.

[CR27] Zou G (2004). A modified poisson regression approach to prospective studies with binary data. American Journal of Epidemiology.

